# Improved method for prioritization of disease associated lncRNAs based on ceRNA theory and functional genomics data

**DOI:** 10.18632/oncotarget.13964

**Published:** 2016-12-15

**Authors:** Peng Wang, Qiuyan Guo, Yue Gao, Hui Zhi, Yan Zhang, Yue Liu, Jizhou Zhang, Ming Yue, Maoni Guo, Shangwei Ning, Guangmei Zhang, Xia Li

**Affiliations:** ^1^ College of Bioinformatics Science and Technology, Harbin Medical University, Harbin, China; ^2^ The First Affiliated Hospital, Harbin Medical University, Harbin, China; ^3^ Key Laboratory of Cardiovascular Medicine Research, Harbin Medical University, Ministry of Education, China

**Keywords:** long non-coding RNA, competing endogenous RNA, functional genomics, prognostic biomarker

## Abstract

Although several computational models that predict disease-associated lncRNAs (long non-coding RNAs) exist, only a limited number of disease-associated lncRNAs are known. In this study, we mapped lncRNAs to their functional genomics context using competing endogenous RNAs (ceRNAs) theory. Based on the criteria that similar lncRNAs are likely involved in similar diseases, we proposed a disease lncRNA prioritization method, DisLncPri, to identify novel disease-lncRNA associations. Using a leave-one-out cross validation (LOOCV) strategy, DisLncPri achieved reliable area under curve (AUC) values of 0.89 and 0.87 for the LncRNADisease and Lnc2Cancer datasets that further improved to 0.90 and 0.89 by integrating a multiple rank fusion strategy. We found that DisLncPri had the highest rank enrichment score and AUC value in comparison to several other methods for case studies of alzheimer's disease, ovarian cancer, pancreatic cancer and gastric cancer. Several novel lncRNAs in the top ranks of these diseases were found to be newly verified by relevant databases or reported in recent studies. Prioritization of lncRNAs from a microarray (GSE53622) of oesophageal cancer patients highlighted ENSG00000226029 (top 2), a previously unidentified lncRNA as a potential prognostic biomarker. Our analysis thus indicates that DisLncPri is an excellent tool for identifying lncRNAs that could be novel biomarkers and therapeutic targets in a variety of human diseases.

## INTRODUCTION

In recent years, a large number of non-coding RNAs (ncRNAs) have been identified by large-scale genomic studies. A type of ncRNAs are called microRNAs (miRNAs) that act by destabilizing and repressing target mRNAs post-transcriptionally and are widely studied in several human diseases [1]. In contrast, only a small number of long non-coding (lncRNAs) (> 200 nucleotides in length) have been functionally characterized. Studies have shown that lncRNAs are involved in a wide range of biological functions, such as chromatin modification [2], the regulation of apoptosis and invasion [3] and genomic imprinting [4] as well as in many human diseases including cancers [5, 6].

Although many novel lncRNA-disease associations have been identified by *in vivo* or *in vitro* experimental methods, identifying new lncRNA-disease associations based on large scale experimental studies is expensive, complex and time-consuming. In addition, the lncRNA-disease associations that are stored in the publicly available databases, LncRNADisease [7] and Lnc2Cancer [8] are fewer in number than the existing lncRNAs. Therefore, there is a need to develop better bioinformatic methods that accurately predict potential lncRNA-disease associations and analyze lncRNA functions in humans.

Several bioinformatic methods have been used to predict novel lncRNA-disease associations. Based on lncRNA expression profiles, Chen and others proposed a predicting method called LRLSLDA [9]. By integrating information from lncRNA and gene expression profiles, Liu and colleagues developed a computational framework to infer human disease-associated lncRNAs [10]. Although these methods increased the efficiency of disease-lncRNA discovery, their results varied tremendously depending on the type of expression data that was being analyzed due to spatio-temporal specificity of lncRNAs. Further, Yang and colleagues proposed a bipartite network based method for analysis of lncRNA-disease associations [11]. Zhou and others prioritized candidate disease-related lncRNAs by walking on the heterogeneous lncRNA and disease network [12]. Although network-based methods provided a functional view to study disease risk lncRNAs, the methodology evaluating functional similarity is time-consuming when large networks are analyzed. Considering the limitations of traditional network methods, Chen and others developed the IRWRLDA method which relies on lncRNA expression and disease semantic similarity [13]. Recently, an integrated method, named KATZLDA was developed to uncover potential lncRNA-disease associations by integrating known lncRNA-disease associations, lncRNA expression profiles, lncRNA functional similarity, disease semantic similarity, and Gaussian interaction profile kernel similarity [14]. Although this method provides a comprehensive ranked list of lncRNAs based on heterogeneous datasets, only few lncRNA candidates are well-annotated. Chen and others proposed a novel calculation model called LNCSIM, that measures the similarity between two lncRNA-associated disease groups and quantifies the functional similarity of each lncRNA pair [15]. Further, Chen and colleagues developed two improved methods, ILNCSIM [16] and FMLNCSIM [17], to predict candidate disease associated lncRNAs based on the assumption that lncRNAs with similar biological functions are involved in similar diseases. These methods have provided valuable results for studying the pathological roles of lncRNAs. However, experimentally confirmed disease-lncRNA associations are limited [18]. Also, developing new and effective methods by integrating data from multiple sources to predict potential disease risk lncRNAs is challenging.

Recent studies have reported that lncRNAs function as competing endogenous RNAs (ceRNAs) and compete with other RNA transcripts [19–21]. By sharing common miRNA-binding sites with mRNAs, lncRNAs can compete with other genes for miRNA molecules, thereby relieving miRNA-mediated target repression. This type of lncRNA-associated ceRNAs have been widely observed in human diseases [22]. Recently, several studies have performed large scale transcriptional analysis to identify potential lncRNA associated ceRNA interactions and further investigate lncRNA functions based on experimental and RNA sequencing data. For example, starBase v.2.0 applies ceRNA function web tools to predict the function of ncRNAs and provides Pan-Cancer ceRNA networks [23]. Another lncRNA-associated ceRNA database, lnCeDB, provides tissue-specific information on ceRNAs [24]. LncACTdb identifies functional lncRNA-miRNA-mRNA interactions through an integrated pipeline and indicates potential cancer prognostic ceRNA biomarkers [25]. Such studies help infer lncRNAs functions and their regulation in diverse human diseases [26]. However, very few studies that predict lncRNA-disease associations based on ceRNA theory exist. The ceRNA theory can improve current disease lncRNA prediction methods by evaluating lncRNA similarities through functional genomics data and bring new insights into ceRNA regulation in diseases.

In this study, our aim was to develop an improved disease associated lncRNA prioritization method named DisLncPri that integrated both ceRNA theory and functional genomics data. Our comprehensive analysis shows that the DisLncPri method helps not only in improving the understanding of lncRNAs regulation at the transcriptional level, but also result in novel biomarker discovery and therapeutic development of disease.

## RESULTS

### Systematic analysis of the functional similarity of disease-associated lncRNAs using ceRNA theory

**Figure 1:**
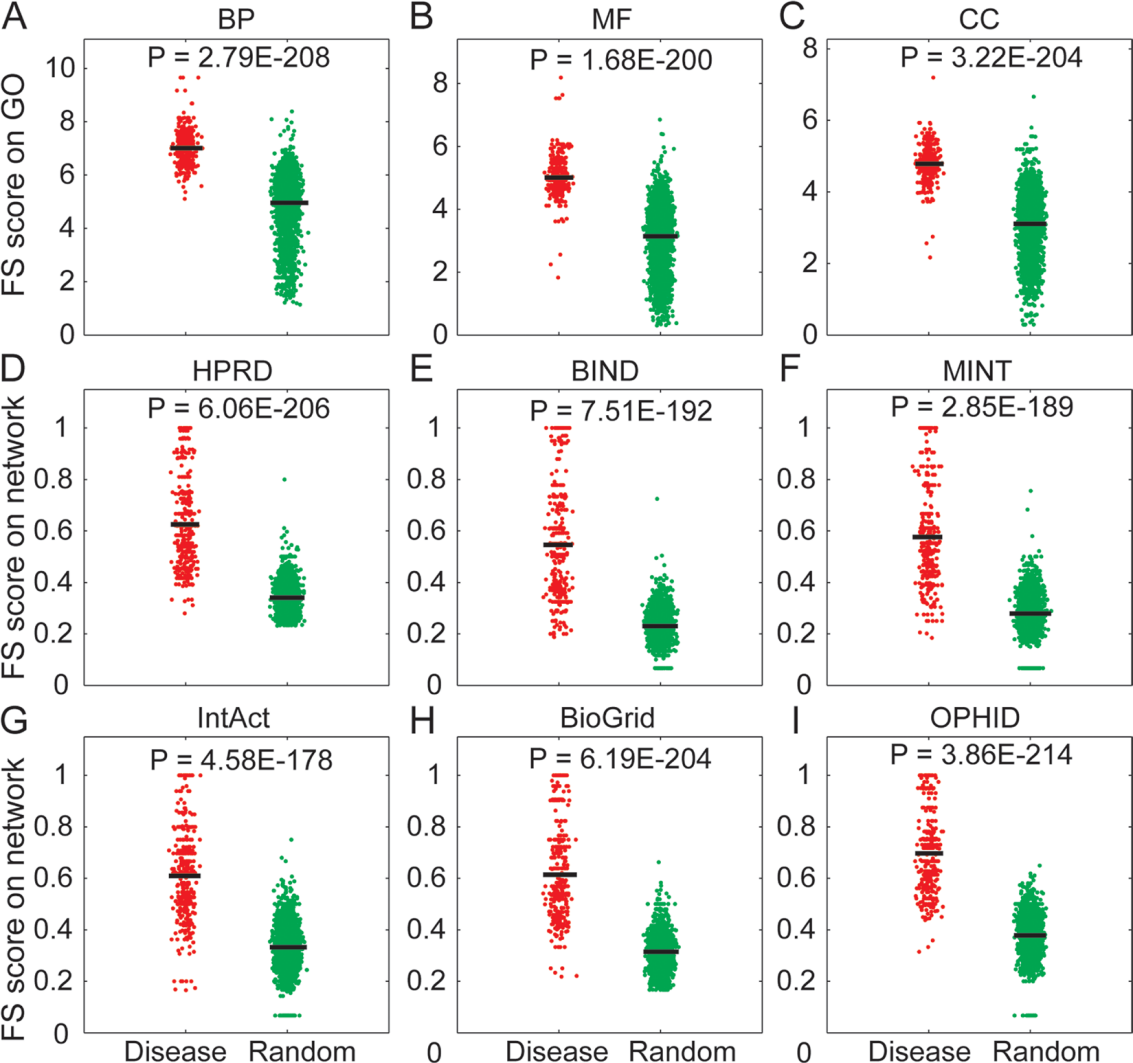
Systematic analysis of the functional similarity for known disease-associated lncRNAs (**A**–**C**) Comparison of FS scores between experimentally validated disease lncRNAs (red points) and randomly selected lncRNAs (green points) based on three orthogonal ontologies of GO. (**D**–**I**) Comparison of FS scores between experimentally validated disease lncRNAs (red points) and randomly selected lncRNAs (green points) based on six biological networks. Experimentally validated disease lncRNA groups had significant higher FS score than random groups. The horizontal bars indicate the mean FS score.

In this study, we used the ‘guilt-by-association’ strategy to identify lncRNAs based on the ceRNA interactions with their competing mRNA partners [27–29]. This strategy had been used in our previous work [25] and other web servers like Linc2GO [30] and starBaseV2.0 [23]. We identified the lncRNA-mRNA ceRNA pairs by an integrated pipeline and experimentally validated the disease-associated lncRNAs from the LncRNADisease database. Through its competing mRNAs, each disease-associated lncRNA was mapped to the functional GO terms from three orthogonal ontologies (BP, MF and CC). For a disease having ‘n’ associated lncRNAs (n≥2), we randomly generated a set of ‘n’ lncRNAs and calculated the functional similarity (FS) score between each of the lncRNA pairs in the validated and the random groups, respectively. The FS score indicates the functional similarity between two gene products by combining the semantic similarities of their associated terms [31]. We found that experimentally validated disease lncRNA groups had significant higher FS score than random groups in each of the three orthogonal ontologies (Figure 1A–1C, Mann-Whitney *U*-test), indicating a high functional similarity between disease-associated lncRNAs.

Based on this strategy, we performed functional analysis on five types of biological networks (HPRD, BIND, MINT, BioGrid and IntAct) and an integrated network (OPHID). The lncRNAs were mapped to the biological network through their respective competing mRNA products. The lncRNAs were treated as nodes within a large undirected graph and the FS score of these nodes was determined. As previously observed for the GO analysis, we found that experimentally validated disease lncRNA groups had a higher FS score than the random groups in each of the network (Figure 1D–1I, Mann-Whitney *U*-test), indicating that the disease lncRNAs were close to each other. Previous studies have indicated that different lncRNAs might have similar functions by performing synergistic regulation in the same network module [25] or functional cluster [32]. Based on these observations, we concluded that lncRNAs associated with the same disease were involved in similiar biological functions.

### Development of DisLncPri

**Figure 2:**
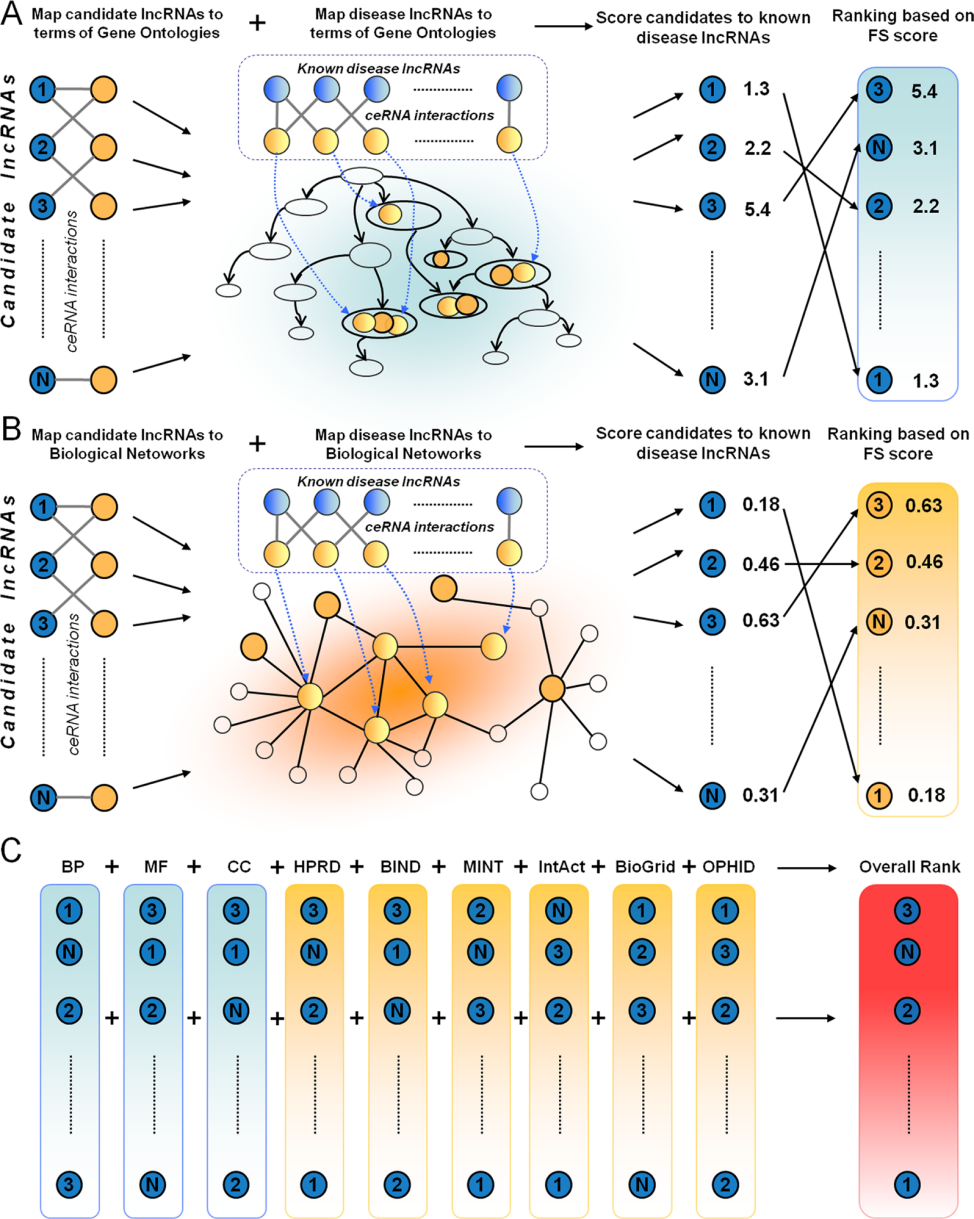
A flowchart of DisLncPri There are three major steps in DisLncPri: (**A**) The candidate lncRNA list is ranked according to their FS score with known seed disease lncRNAs based on the three orthogonal function ontologies of GO. (**B**) With a similar strategy as in step A, the candidate lncRNA list is ranked according to their FS score based on the context of six biological networks. (**C**) The nine ranked lncRNA lists from steps (A and B) are combined for each candidate lncRNA into a single list using multiple rank fusion method. LncRNAs are indicated as blue circles and mRNAs are indicated as yellow circles.

Based on the above analysis, we hypothesized that the property of functional similarity could be used as an advantage in prioritization of candidate disease related lncRNAs and developed the DisLncPri method (Figure 2). Through their competing mRNAs, lncRNAs were mapped to the functional context such as GO terms and biological network. There were three major steps in DisLncPri. In the first step, known disease-associated lncRNAs (seed lncRNAs) and candidate lncRNAs were mapped to three orthogonal function ontologies (BP, MF and CC) of GO (Figure 2A). For every candidate lncRNA, the average FS scores were calculated between the candidate and the seed group based on the GO function and the candidate lncRNA was ranked according to the FS. In the second step, the seed lncRNAs and candidate lncRNAs were mapped to six biological networks and the average FS score was calculated for each candidate based on the network and ranked accordingly (Figure 2B). In the third step, the nine ranked lncRNA lists from the previous two steps were combined for each candidate lncRNA into a single list using multiple rank fusion method (Figure 2C). For each rank, the Q statistic method generated an integrated score. This rank indicated the overall priority for each candidate lncRNA.

### Performance of DisLncPri

**Figure 3:**
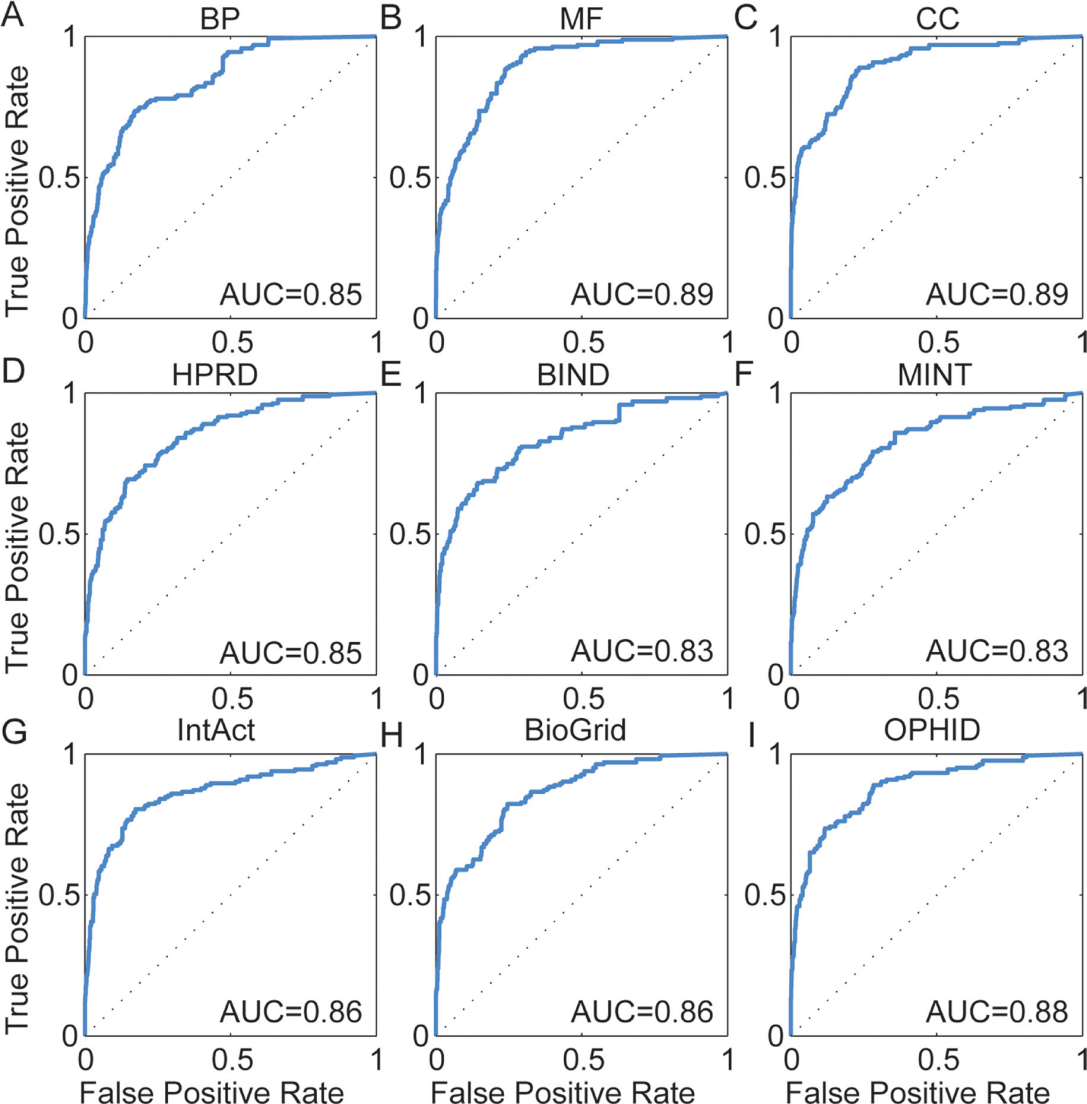
ROC curves for LOOCV analysis (**A**–**C**) Three orthogonal ontologies of GO. (**D**–**I**) Six biological networks. DisLncPri achieved a reliable AUC value from 0.83 to 0.89.

To assess the ability of DisLncPri to recognize experimentally validated lncRNAs of corresponding diseases, we performed a large scale leave-one-out cross validation (LOOCV) analysis based on experimentally verified disease-lncRNA associations from the LncRNADisease database [7]. We calculated sensitivity (frequency of testing lncRNAs that were ranked above a particular cut-off point) and specificity (the percentage of lncRNAs ranked below the cut-off point) for the rank positions. Then, we plotted receiver operating characteristic (ROC) curves based on the functional properties of the testing lncRNAs to facilitate the comparison between different functional genomics data (Figure 3). The area under curve (AUC) value was then measured to evaluate algorithm performance. AUC value of 1.0 suggests that the lncRNA being tested is ranked on top whereas a value of 0.5 indicates that the lncRNA being tested is randomly ranked along the list. For every functional genomics dataset tested, DisLncPri achieved a very reliable AUC value ranging from 0.83 to 0.89 (Figure 3A–3I). This indicated that DisLncPri was a sensitive and specific method of ranking known disease lncRNAs regardless of the data source used.

We further tested the stability of DisLncPri by performing the LOOCV analysis on the Lnc2Cancer database that we had previously developed [8]. For each functional genomics data source, DisLncPri achieved a reliable AUC value ranging from 0.72 to 0.88 (Supplementary Figure S1). These results showed that the DisLncPri method was efficient in recovering known disease-lncRNA associations from a candidate disease-related lncRNA set.

### Improvement of DisLncPri

**Figure 4:**
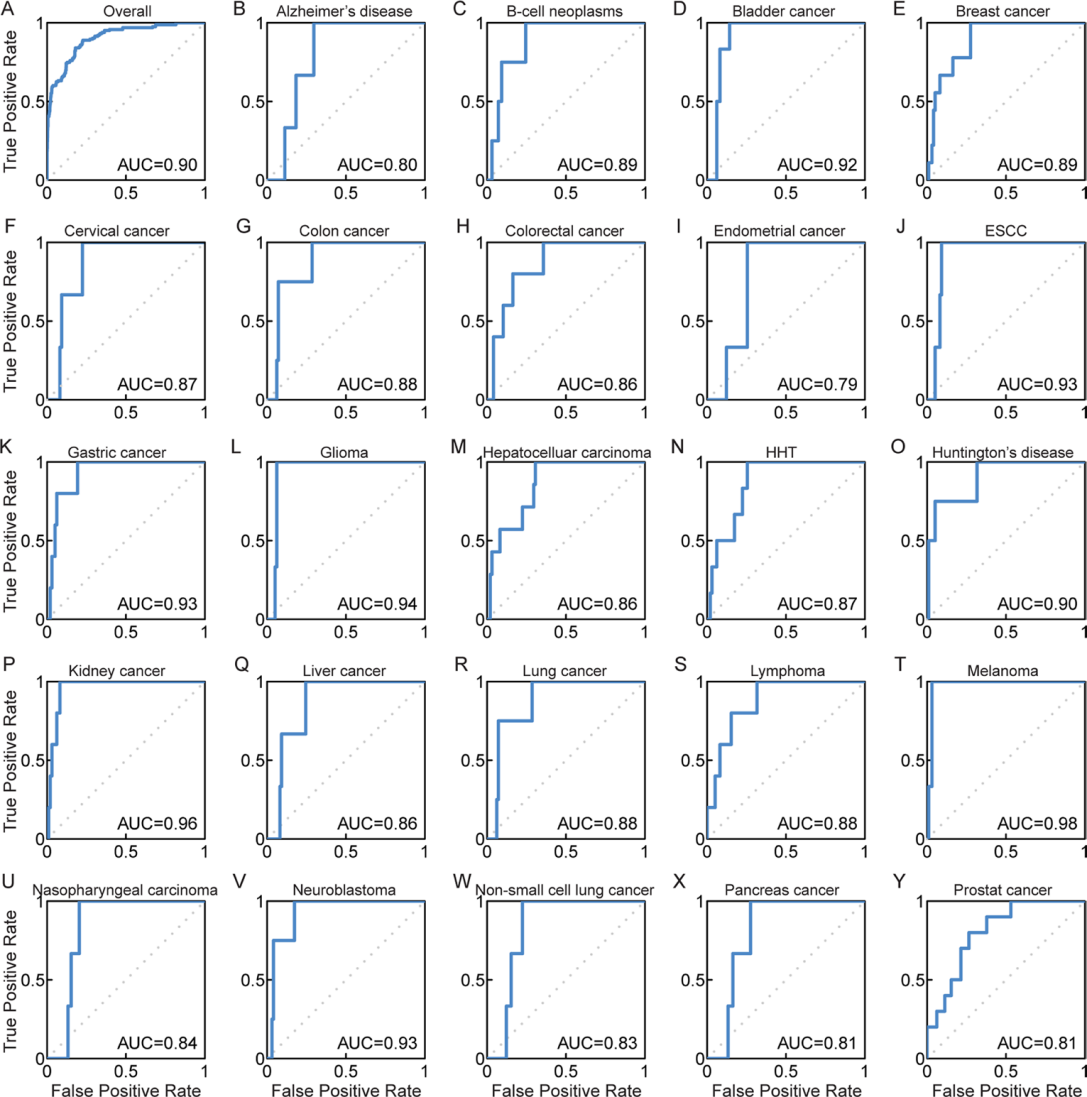
ROC curves for DisLncPri by integrating different functional genomics dataset (**A**) The overall ROC curve yielded the highest AUC value of 0.90. (**B**–**Y**) Case studies for 24 complex diseases in LOOCV analysis after improvement of DisLncPri. HTT: Hereditary Haemorrhagic Telangiectasia.

Although the disease lncRNAs that were tested ranked highly in the candidate list, our analysis generated distinct prioritizations for multiple functional genomics data sources. In order to minimize variability and increase ranking performance, previous studies had used an integrating strategy to deal with multiple ranks from heterogeneous data sources [33, 34]. We integrated the Q statistic rank fusion method [33] in the DisLncPri framework to minimize the ranking order discrepancy and improve the prioritization efficiency. We had used this strategy previously to prioritize miRNA target genes [35] and cancer-associated lncRNA-mediated feed-forward loops [36]. Based on this strategy, DisLncPri integrated the nine ranked lists from different functional genomics datasets in LOOCV analysis of LncRNADisease. The final overall list we obtained was better than all other ranks shown in Figure 3 and yielded the highest AUC value of 0.90 (overall ROC plot in Figure 4A), indicating improved efficiency of DisLncPri. We further plotted ROC curves for more than 20 individual diseases based on the LOOCV analysis and obtained highly reliable AUC values for melanoma (0.98), kidney cancer (0.96) and glioma (0.94) as shown in Figures 4B–4Y. When we applied the DisLncPri to integrate the multiple rank list of LOOCV analysis based on Lnc2Cancer dataset, the integrated rank list was better than all the other rank lists and yielded the highest AUC value of 0.89 (Supplementary Figure S2).

### Comparsion with other methods

**Figure 5:**
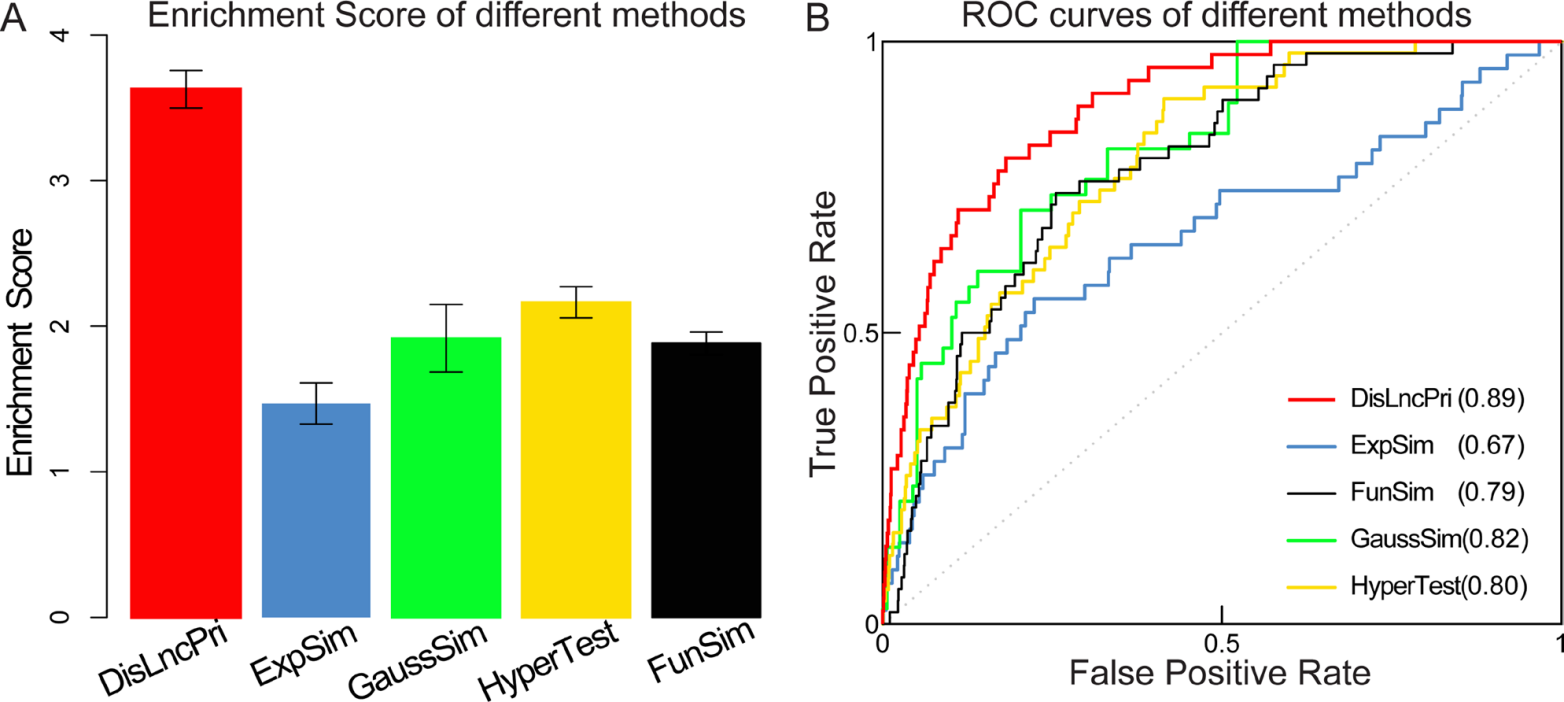
Comparison of DisLncPri analysis with other methods (**A**) DisLncPri method had a higher ES score than other similar methods. Error bars are 95% confidence Interval. (**B**) DisLncPri had the highest AUC value in comparsion with others.

We, then compared DisLncPri to other computational methods that are based on the assumption that similar lncRNAs tend to associate with similar diseases [37]. Several algorithms are used to evaluate similarity between lncRNAs. For example, the expression similarity (ExpSim) algorithm is based on expression profiles [9, 10]; the gaussian interaction profile kernel similarity (GaussSim) algorithm is based on known disease-lncRNA relationships [14, 38]; the functional similarity (FunSim) algorithm is based on the structure of a directed acyclic graph (DAG) in the disease/gene ontology [15] and biological networks [11, 12, 38]. Recent studies have also proposed using the hypergeometric distribution test (HyperTest) algorithm to infer disease-lncRNA [10, 39] and disease-miRNA [40] associations by evaluating the significance of common targets. Our strategy was to prioritize all the candidate lncRNAs for a certain disease using DisLncPri and compare the analysis with the ExpSim, GaussSim, FunSim and HyperTest algorithms that have been used by majority of disease-associated lncRNA prioritization methods. We manually checked the predicted lncRNA lists of different methods to find the rank positions of experimentally verified cases in the literature. We then analyzed the case studies of several high morbidity and mortality diseases like alzheimer's disease, ovarian cancer, pancreatic cancer and gastric cancer (Supplementary Table S1). For each disease, known disease-associated lncRNAs were used as seed lncRNAs, and all the other unknown lncRNAs were used as candidates for prioritization. Since different methods could result in different sized prediction lists, we calculated the enrichment score (ES) values based on the rank positions of experimentally verified disease lncRNAs for comparison [35]. ES value was defined as *log2 (n/2/rank)* for a ranked list of *n* lncRNAs. We found that DisLncPri method had a higher ES score than other similar methods (Figure 5A). Further, performance evaluation was carried out in terms of sensitivity and specificity, and the ROC curves were plotted (Figure 5B). LOOCV analysis was then performed to compare DisLncPri with others. We found that DisLncPri had the highest AUC value (0.90) in the LOOCV analysis (Supplementary Figure S3). The ROC curves for the different diseases are shown in Supplementary Figures S4–S7. The analysis showed that the DisLncPri method had the highest AUC value in comparison to other methods.

### Case studies of human diseases

To demonstrate the ability of our DisLncPri method in predicting novel disease-associated lncRNAs, we tested case studies of four important diseases (alzheimer's disease, ovarian cancer, pancreatic cancer and gastric cancer). Prediction results for the top 20 ranks were manually verified by a literature survey (Table 1). Detailed analysis for each disease is shown below.

**Table 1:** Novel lncRNA-disease associations confirmed by literature survey in the top 20 ranked list of DisLncPri

Disease	lncRNA	Ensembl ID	Rank
Alzheimer's disease	MEG3	ENSG00000214548	1
	PVT1	ENSG00000249859	6
	LINC01616	ENSG00000261340	13
Ovarian cancer	GAS5	ENSG00000234741	1
	MALAT1	ENSG00000251562	4
	MEG3	ENSG00000214548	6
	HOTAIR	ENSG00000228630	9
Pancreatic cancer	GAS5	ENSG00000234741	4
	AP000221.1	ENSG00000229962	11
	CTC-338M12.5	ENSG00000250222	17
Gastric cancer	FRGCA	ENSG00000236663	1
	MALAT1	ENSG00000251562	13
	MEG3	ENSG00000214548	20

Alzheimer's disease (AD) is the most prevalent cause of dementia characterized by progressive loss of memory, cognitive and intellectual capacity [41]. In the DisLncPri predicting results, we found 3 novel lncRNAs in top 20 (MEG3 at 1, PVT1 at 6, and LINC01616 at 13) that were not known to be associated with AD in the LncRNADisease database although being linked to AD in recently reported studies. MEG3 was reported to activate and improve the binding affinity to target gene promoter of TP53 [42], which is a potential biomarker for diagnosis of AD [43]. PVT1 was shown to regulate c-Myc gene transcription over a long distance [44] and the c-Myc pathway is a key player in progression of AD [45]. In a previous study, LINC01616 was downregulated in AD patients compared with controls [46]. Functional analysis indicated that LINC01616 was associated with the protein ubiquitination pathway. The ubiquitin-proteasomal system pathway is altered in AD brains and multiple genes in this pathway have been implicated in AD pathogenesis [47].

Ovarian cancer is the most lethal gynecological cancer reported to metastasize frequently in women globally [48]. In the DisLncPri predicting result list, we found 4 novel lncRNAs in top 20 (GAS5 at 1, MALAT1 at 4, MEG3 at 6 and HOTAIR at 9) that were recently associated with ovarian cancer. GAS5 was shown to be downregulated and characterized to inhibit cell proliferation, migration and invasion and promote apoptosis in epithelial ovarian cancer cells [49]. A recent study found that MALAT1 was deregulated in ovarian cancer and postulated to play a suppressive role [50]. MEG3 was shown to activate p53 and involved in progression of various types of cancers. Ectopic expression of MEG3 was reported to suppress the proliferation and growth of ovarian cancer cells and promote apoptosis [51]. Overexpression of HOTAIR was recently shown to predict poor patient prognosis and promote tumor metastasis in epithelial ovarian cancer [52].

Pancreatic cancer has a high mortality rate and the 5-year relative survival rate is extremely low [53]. The DisLncPri data showed several novel lncRNAs associated with pancreatic cancer. These included GAS5 at 4, AP000221.1 at 11 and CTC-338M12.5 at 17. A previous study identified the crucial role for GAS5 in the molecular etiology of pancreatic cancer and as a potential therapy target [54]. AP000221.1 and CTC-338M12.5 were shown to be differentially expressed in a drug-resistant pancreatic cancer cell line with increasing dosages of gemcitabine [55] suggesting that they may be good diagnostic or prognostic biomarkers as well as therapeutic targets.

Gastric cancer is one of the most commonly diagnosed cancers and the second leading cause of cancer death worldwide [56]. We found 3 novel lncRNAs in the top 20 (FRGCA at 3, MALAT1 at 13 and MEG3 at 20) that were recently associated with gastric cancer. Knockdown and overexpression experiments of FRGCA showed that it played a critical role in gastric cancer progression and was a potential therapeutic target [57]. MALAT1 was found to be highly expressed in gastric cancer cells and probably promoted GC cell proliferation partly by regulating SF2/ASF [58]. MEG3 was identified as a competing endogenous RNA to regulate gastric cancer progression and ectopic expression of MEG3 in HGC-27 and MGC-803 cells was shown to inhibit cell proliferation, migration, invasion, and promote apoptosis [59].

### Analysis of high-throughput datasets

**Figure 6:**
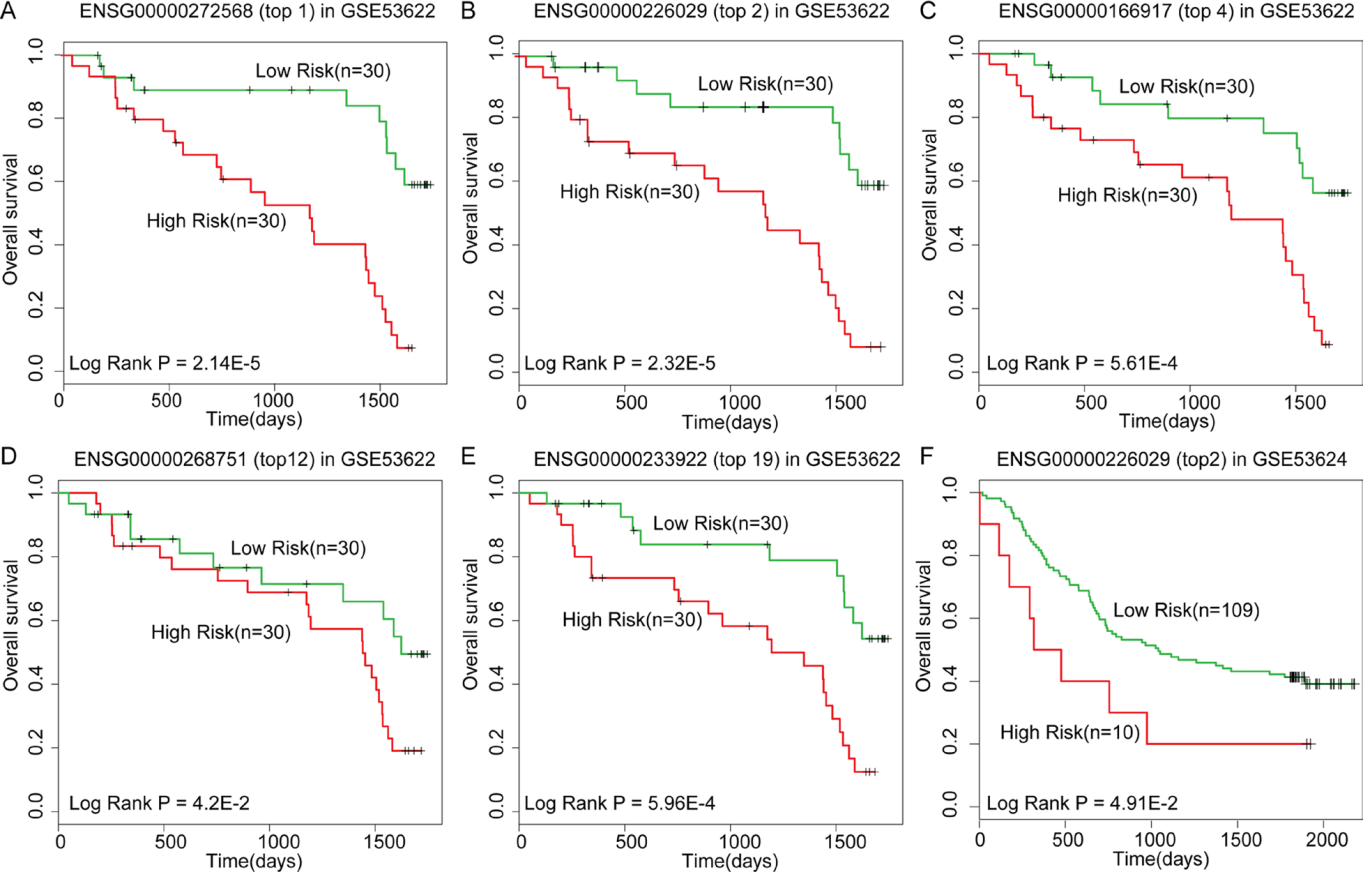
Kaplan-Meier survival analysis for lncRNAs as predicted by DisLncPri (**A**–**E**) DisLncPri predicted five risk lncRNAs which could significantly divide the 60 OSCC patients into two groups with high- and low-survival rates. (**F**) The lncRNA ENSG00000226029 (top 2 in the list) had significant effects on OSCC patient survival in another independent dataset.

High throughput microarray and RNA sequencing analysis are generally performed to obtain whole-transcriptome sequences and detect the less-abundant mRNA and lncRNA transcripts in disease and paired normal samples. The drawback of these analyses is the large amount of differently expressed mRNAs/lncRNAs obtained that needs to be validated to eliminate false positives before any biological analysis [60]. For example, oesophageal cancer is one of the most deadly forms of disease worldwide. In China, over 90% of the oesophageal cancer cases are oesophageal squamous cell carcinoma (OSCC) that is highly aggressive and malignant with poor prognosis [61]. A recently published dataset (GSE53622) provided lncRNA expression profile of OSCC and adjacent normal tissues from 60 patients. In this dataset, 1834 differentially expressed lncRNAs were found at the threshold of 0.05 *p*-value (Bonferroni corrected Student's t test) and 980 differentially expressed lncRNAs at a stringent threshold of 0.01. In such a scenario, it is hard to choose appropriate candidates for further biological analysis. In order to reduce the false positives, we used DisLncPri to prioritize the lncRNA lists resulting from the expression profile of GSE53622. Three well-annotated lncRNAs (H19, HOTAIR and ANRIL) from lncRNADisease database were used as known OSCC-related seed lncRNAs. These three lncRNAs are associated with prognosis of OSCC and other cancers [62–64]. Subsequently, we examined whether the top lncRNAs prioritized by DisLncPri were related with OSCC patient prognosis. We performed univariate Cox regression analysis on the top 20 prioritized lncRNAs based on their expression value across 60 OSCC patients. We found 8 lncRNAs that had significant effects on OSCC patient survival (Table 2, P < 0.05). To evaluate the association between each of the 8 lncRNAs with OSCC prognosis, we performed Kaplan-Meier survival analysis and found 5 that divided the 60 OSCC patients into two groups with either high- and low-survival rates (Figure 6A–6E). To further test whether these lncRNAs could be used as potential prognostic biomarkers, we applied them to an independent OSCC dataset (GSE53624) that had 119 patients with well-annotated clinical follow-up data. We found lncRNA ENSG00000226029 (top 2 in the list) that had significant effects on OSCC patient survival in the independent dataset (*P* = 0.03, Coefficient = 2.43). Kaplan-Meier survival analysis revealed that this lncRNA also divided the 119 OSCC patients into high- and low-risk groups with significantly different survival times (Figure 6F, P < 0.05). To the best of our knowledge, lncRNA ENSG00000226029 has not been reported to be related with OSCC in previous studies. Thus, our analysis identified lncRNA ENSG00000226029 as a novel OSCC risk lncRNA that could be used as a novel prognostic biomarker.

**Table 2:** Univariate Cox regression analysis showing 8 lncRNAs that significantly affect OSCC patient survival (*P* < 0.05)

Rank	LncRNAs	Ensembl ID	HR(95%CI)	Coefficient	*P*-value
1	CTB-113D17.1	ENSG00000272568	5.20(2.51–10.75)	1.65	8.89E–06
2	RP4-798A10.2	ENSG00000226029	9.75(3.59–26.49)	2.28	7.93E–06
4	MIR202HG	ENSG00000166917	6.05(2.13–17.14)	1.80	7.08E–04
6	TFAP2A-AS1	ENSG00000229950	0.57(0.37–0.88)	−0.56	1.10E–02
12	SCGB1B2P	ENSG00000268751	0.23(0.07–0.79)	−1.47	1.96E–02
13	RP11-510M2.2	ENSG00000247324	1.91(1.02–3.59)	0.65	4.32E–02
19	AL133493.2	ENSG00000233922	0.17(0.06–0.48)	−1.78	9.10E–04
20	MALAT1	ENSG00000251562	2.90(1.11–7.60)	1.07	3.02E–02

## DISCUSSION

In our previous study, we performed a framework to prioritize cancer risk miRNAs using GO data only [65]. Further, we developed a miRNA target prioritization method by integrating biological process of GO and the HPRD network [35]. Although successful in context of miRNAs, this framework did not highlight contributions from other functional data sets. In this work, we proposed an improved disease associated lncRNA prioritization method named DisLncPri in which lncRNAs were mapped to nine functional genomics contexts through their mRNA interactions. We fused multiple functional data sets and used Q statistic method to integrate distinct functional correlation prioritization ranks into a single rank. This strategy was used to discover the missing annotated lncRNAs and minimize bias for well-annotated cases.

We then performed systematic analysis to infer lncRNA relationships and found high functional similarity between experimentally validated lncRNAs of the same disease. To test whether this functional similarity could be used as an advantage in prioritization of candidated disease related lncRNAs, we performed a large scale leave-one-out cross validation strategy across different functional contexts. Our method achieved highly AUC values up to 0.90 and 0.89 for the LncRNADisease [7] and lnc2Cancer [8] datasets, respectively. Then, we performed case studies of high morbidity and mortality diseases like alzheimer's disease, ovarian cancer, pancreatic cancer and gastric cancer (Supplementary Table S1). We manually checked the predicted lncRNA lists of different methods and found that several novel lncRNAs in the top rank were newly verified by relevant databases or in recent experimental studies.

The major drawback of high-throughput analyses is the large amount of differentially expressed genes that requires extensive validation analysis before performing biological experiments [60]. To reduce the false positive lncRNAs from disease-control microarray analysis, we used DisLncPri to prioritize lncRNAs from a microarray (GSE53622) of oesophageal cancer. In the list of top 20, we found eight lncRNAs that were significantly associated with the prognosis of OSCC patients. Survival analyses demonstrated that lncRNA ENSG00000226029 (top 2) also had significant effects on OSCC patient survival in an independent dataset (GSE53624) and therefore can be a key prognostic biomarker for OSCC. Our analysis therefore suggests that the DisLncPri method not only will improve the understanding of lncRNA-disease associations, but also help discover and authenticate novel biomarkers and therapeutics.

## MATERIALS AND METHODS

### Identification of potential ceRNA interactions

Putative miRNA-lncRNA interactions were identified using miRanda algorithm (version Nov, 2010) with default parameters (Score ≥140 and Energy≤7.0) [29]. The human mature miRNA sequences were downloaded from the miRBase (release 21) [66]. The lncRNA sequences were obtained from the GENCODE database (v21) [67]. A total of 15877 lncRNAs were used as candidates in our framework. Furthermore, the AGO-CLIP sequencing datasets [23] were used to identify experimentally supported cases from the set of predicted miRNA-lncRNA interactions. A total of 1007618 unique binding site clusters were compiled in humans. The miRNA-mRNA interactions were downloaded from two highly reliable online miRNA reference databases—the TarBase (v6) [68] and the mirTarBase (release 4.5) [69], which store manually curated collections of experimentally supported miRNA targets. After combining datasets, 43497 validated non-redundant human miRNA-target pairs were assembled for this study.

To identify the lncRNA-mRNA ceRNAs, a hypergeometric test was used to evaluate the significance of the shared common miRNAs between each lncRNA and mRNA. If the genome context had a total number of ‘*N*’ miRNAs, of which ‘*K*’ and ‘*M*’ are the numbers of miRNAs associated with the current lncRNA and mRNA, respectively and ‘*x*’ is the common miRNA number shared by the lncRNA and mRNA, the *P* value was calculated as follows. An adjusted *p* < 0.01 by Benjamini and Hochberg correction was used as the threshold.

$$P=1-\sum_{t=0}^{x}\frac{\left(\begin{array}{c} K\\ t \end{array}\right)\left(\begin{array}{c} N-K\\ M-t \end{array}\right)}{\left(\begin{array}{c} N\\ M \end{array}\right)},$$ (1)

### Leave-one-out-cross validation

To test the performance of DisLncPri, we carried out a large scale LOOCV analysis based on experimentally verified disease-lncRNA associations. For a given disease with a number of ‘*n*’ experimentally verified lncRNAs, these ‘*n*’ lncRNAs were used as seed sets. In each validation run, we selected an lncRNA as the test case from the ‘*n*’ seed lncRNAs one by one. Further, the test case lncRNA was deleted from seed sets and added to 99 randomly generated lncRNAs without any reported association with the analyzed disease. This group of 100 lncRNAs was used as the candidate set. DisLncPri then used the ‘*n-1*’ seed lncRNAs to prioritize the 100 candidate lncRNAs (including the 1 test case) based on their average FS scores with seed lncRNAs. We localized the rank position of the test case in each validation run. These procedures were applied to each of the nine functional genomics data (three orthogonal ontologies of GO and six biological networks).

### Functional similarity score

For a given disease with ‘*n’* known lncRNAs (*dl-_1_,... ,dl _j_,...,dl _n_*) and a set of ‘*m’* candidate lncRNAs (*cl _1_,... ,cl _i_,...,cl _n_*), the FS values between each ‘*cl’* and *‘dl’* pair were calculated based on the context of GO and biological network, respectively. For each of the candidate lncRNAs, a number of ‘*n* FS’ were generated and the average of all the ‘*n* FS’ was calculated as final FS score. Further, the candidate lncRNAs were ranked based on the final FS (Supplementary Figure S8).

For a candidate lncRNA having ‘*m’* competing genes (*clg_1_,…,clg_i_…clg_m_*) and a known disease lncRNA having ‘*n’* competing genes (*dlg_1_,…,dlg_i_…dlg_n_*), the FS score between these two lncRNAs can be calculated as the average value of ‘*m* x *n* FS’ scores between each gene pairs:

$$FS=\sum_{i=1}^{m}\sum_{j=1}^{n}FS_{ij}/(m\times n),$$ (2)

In the context of GO annotation, the FS score between two genes was previously defined [70] and used as the information content (IC) value of the most informative common ancestor among the terms mapped (Supplementary Figure S9). For two genes *i* and *j*, with GO terms, *a* and *d* as their common ancestors, the IC values for *a* and *d* terms were calculated as:

IC(a)=−lognaN,IC(d)=−logndN, (3)

‘*n*a*’* is the number of genes mapped to term ‘*a’*, ‘*n*d*’* is the number of genes mapped to term ‘*d’*, and ‘*N’* is the total number of genes in the whole human genome. The *FS* score for two genes ‘*i’* and ‘*j’* is defined as *max(IC (a), IC (d))*, which is the most informative common ancestor term of ‘*i* ‘and ‘*j’*.

As previous studies have indicated that functionally related biological molecules tend to be implicated in the same network module or close to each other, the functional similarity for the two nodes can be evaluated by their topological relationship [12, 35]. In context of the biological network, the FS score between two gene nodes was defined as the reciprocal of shortest path (Supplementary Figure S10). A short path between two gene nodes will lead to a higher FS score thus indicating high functional similarity. Dijkstra's algorithm was used to calculate the shortest path between two nodes in the network.

### Multiple data rank fusion

We calculated the overall ranks from separate lncRNA lists using the following *Q* statistic formula, used in previous multiple rank fusion studies [33, 35]:

$$Q(r_{1},r_{2},\ldots r_{N})=V_{N}N!,V_{0}=1,V_{k}=\sum_{i=1}^{k}{\left(-1\right)}^{i-1}\frac{V_{k-i}}{i!}r_{N-k+1}^{i},$$ (4)

where *r*_1_ is the rank ratio for data source ‘*i*’, *N* is the number of data sources used and *r*_0_ = 0.

### Survival analysis

Univariate Cox regression analysis was used to identify lncRNAs that significantly impacted patient survival (*P* < 0.05). The Kaplan-Meier survival analysis was performed for the two groups of patients and statistical significance was assessed using the log-rank test (*P* < 0.05). All analyses were performed based on R 3.2.2 framework.

### Biological network datasets

We downloaded biological datasets from six databases: HPRD (v9.0) [71], BIND (v1.0) [72], MINT (v2.5) [73], BioGrid (v3.1.90 ) [74], IntAct (v2.0) [75] and OPHID (v1.95) [76]. To deal with the network redundancy, self-loops of one node and round-trips between two nodes were refined into one interaction. Detailed information of nodes and interactions are shown in Supplementary Table S2. Cytoscape software (v3.1.1) was used to illustrate and analyze the properties of the networks (Supplementary Figures S11A–S11F and S12A–S12F).

### LncRNA expression profiles

The genome-wide lncRNA expression profiles across different human tissues were derived from the NONCODE database [77] that contains 16 tissues of the HumanBodyMap 2.0 data (ENA archive: ERP000546) and eight cell lines (GSE30554). LncRNA expression files of two independent oesophageal cancer datasets (GSE53622, GSE53624) were downloaded from the GEO database. The probe sets were re-annotated to the human genome by BLAST method with alignment score of 100% identity. The mean expression of the different array probes was calculated to infer their expression levels. Patients with well-annotated clinical follow-up information were retained for survival analysis.

### LncRNA-disease associations

Known lncRNA-disease associations were downloaded from the LncRNADisease and the Lnc2Cancer databases. After deleting duplicate records and mapping lncRNA name to Ensembl ID, we found 453 distinct experimentally supported lncRNA-disease associations for 171 lncRNAs and 182 diseases in LncRNADisease database and 625 distinct experimentally supported lncRNA-disease associations for 295 lncRNAs and 87 cancers in Lnc2Cancer database.

## SUPPLEMENTARY MATERIALS FIGURES AND TABLES




