# Supplementary material for: Improved method for prioritization of disease associated lncRNAs based on ceRNA theory and functional genomics data
Source: Oncotarget. 2016 Dec 15;8(3):4642–55. doi: 10.18632/oncotarget.13964 (PMC5354861; doi:10.18632/oncotarget.13964)
Supplement: Supplementary file 1 [file oncotarget-08-4642-s001.pdf]

Improved method for prioritization of disease associated lncRNAs based on ceRNA theory and functional genomics data

Supplementary Materials

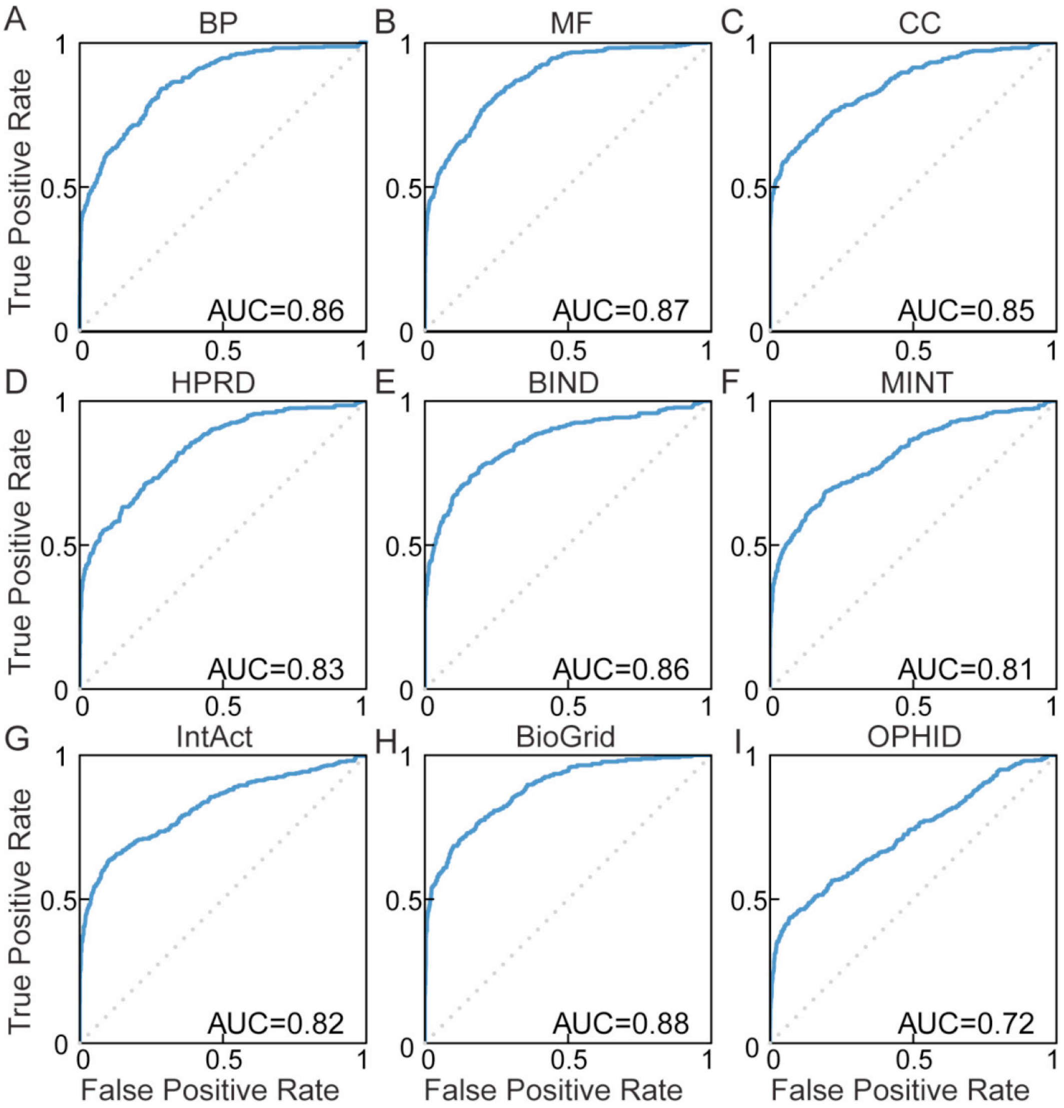

Supplementary Figure S1: ROC curves for LOOCV analysis using known disease-lncRNA associations from Lnc2Cancer database. (**A–C**) Three orthogonal ontologies of GO. (**D–I**) Six biological networks. DisLncPri achieved a reliable AUC value from 0.72 to 0.88.

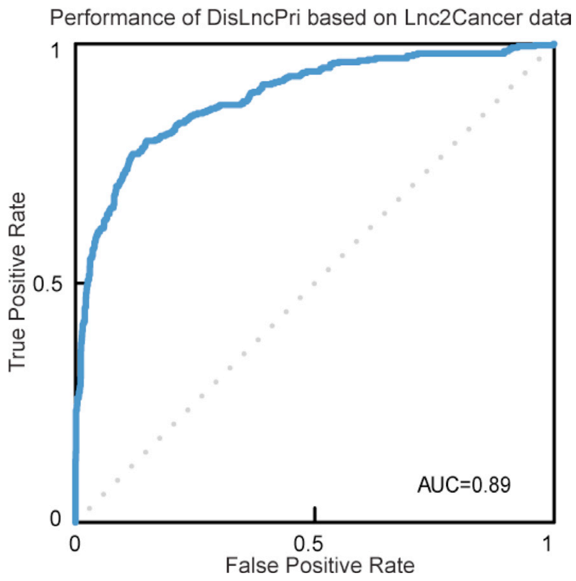

Supplementary Figure S2: ROC curves for DisLncPri by integrating different functional genomics dataset. The overall ROC curve yielded AUC value of 0.89 for the Lnc2Cancer dataset.

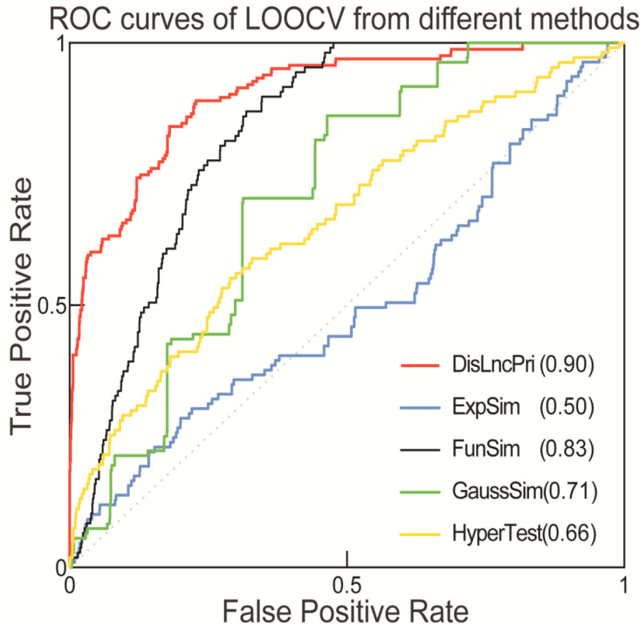

Supplementary Figure S3: ROC curves for LOOCV analysis comparing different methods. DisLncPri had the highest AUC value of 0.90 than others (0.50–0.83).

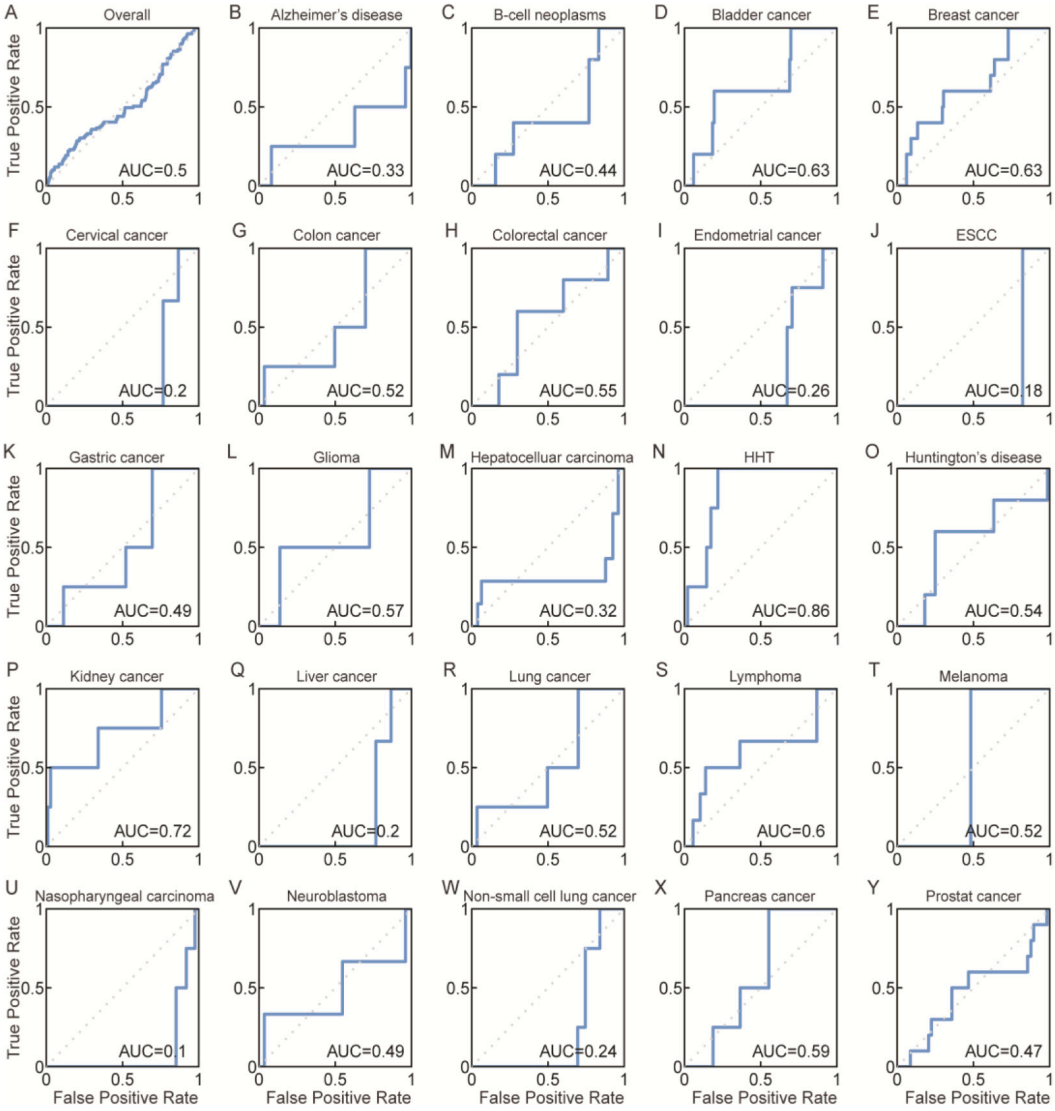

Supplementary Figure S4: ROC curves and AUC values for ExpSim method. (**A**) The overall ROC curve of ExpSim was 0.50. (**B–Y**) Case studies for 24 complex diseases in LOOCV analysis. HTT: Hereditary Haemorrhagic Telangiectasia.

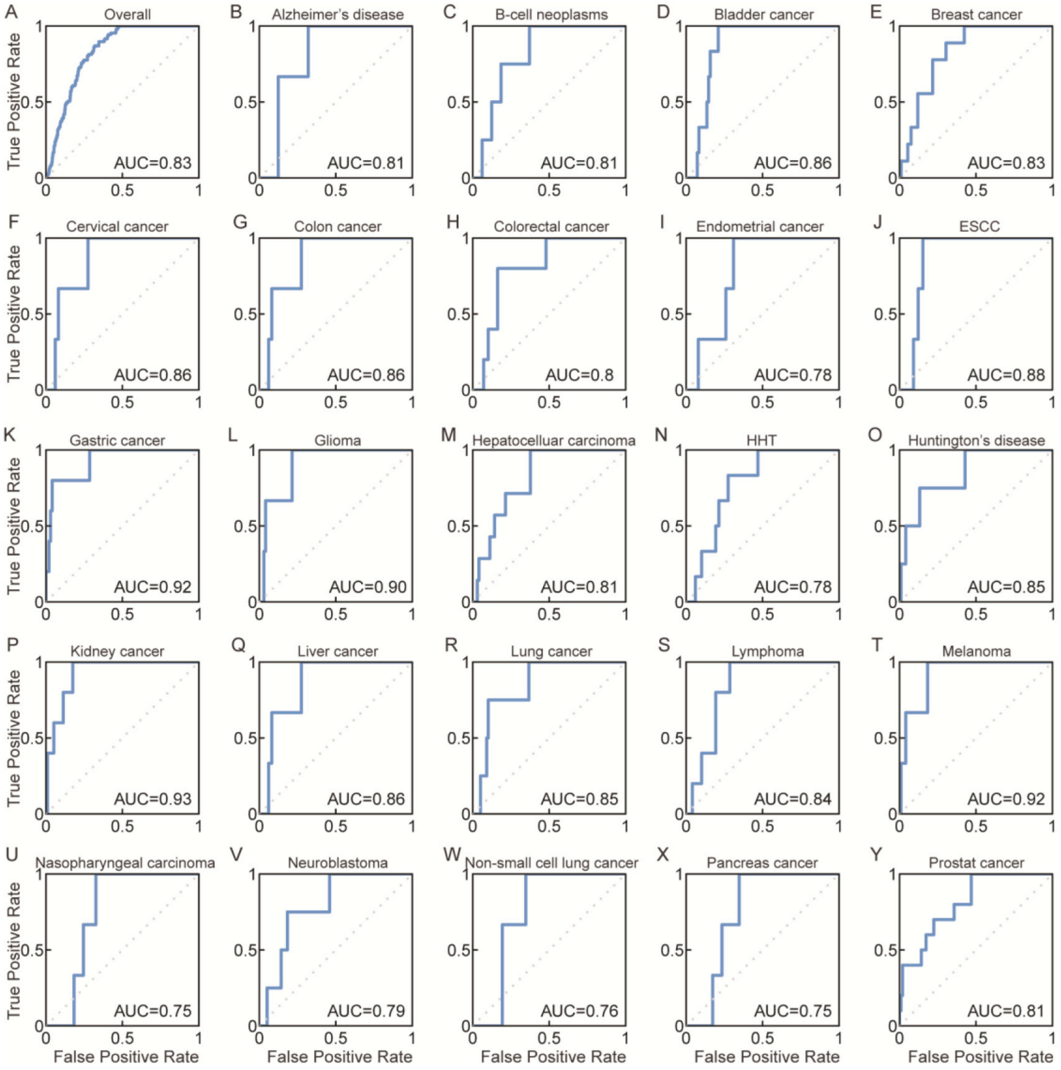

Supplementary Figure S5: ROC curves and AUC values for FunSim method. (**A**) The overall ROC curve of ExpSim was 0.83. (**B**–**Y**) Case studies for 24 complex diseases in LOOCV analysis. HTT: Hereditary Haemorrhagic Telangiectasia.

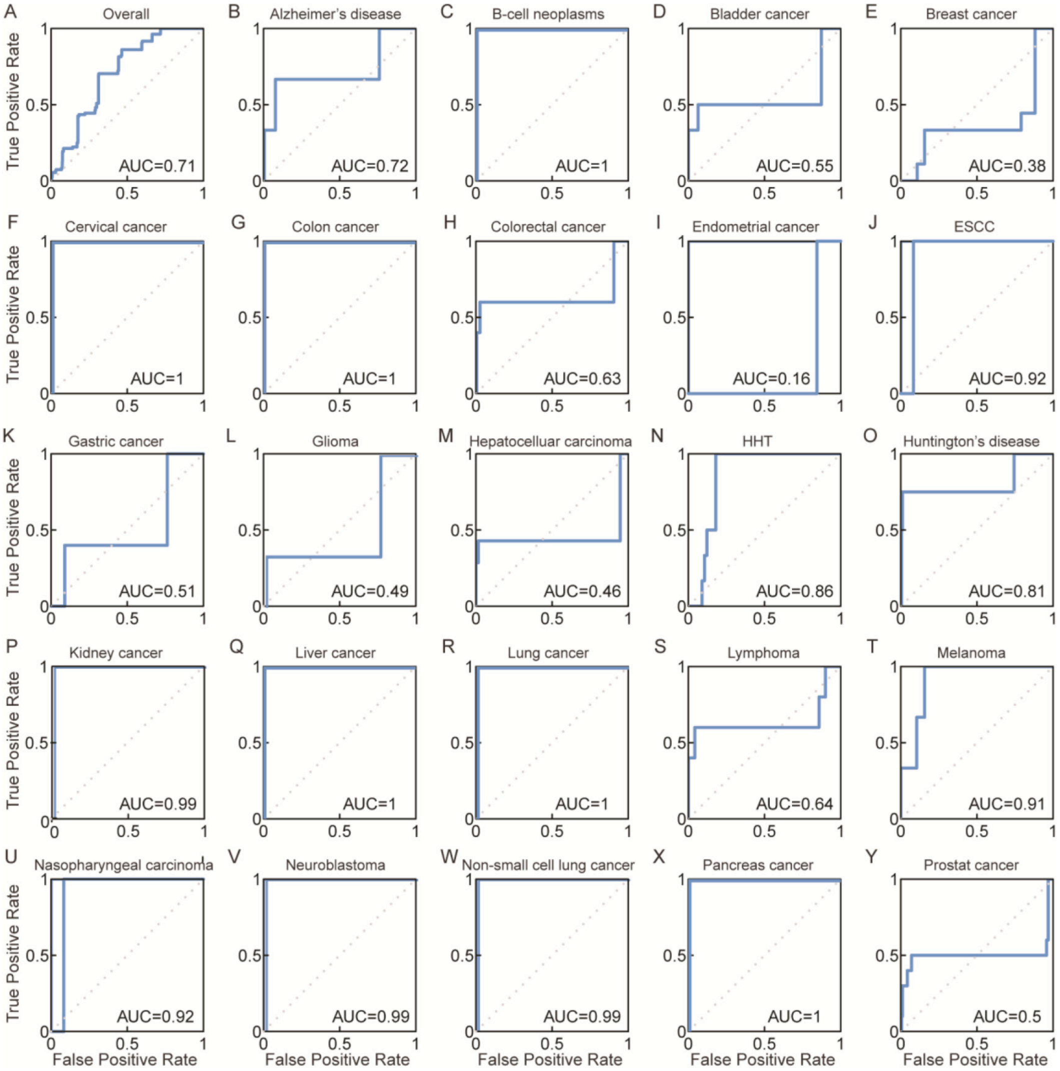

Supplementary Figure S6: ROC curves and AUC values for GaussSim method. (**A**) The overall ROC curve of ExpSim was 0.71. (**B–Y**) Case studies for 24 complex diseases in LOOCV analysis. HTT: Hereditary Haemorrhagic Telangiectasia.

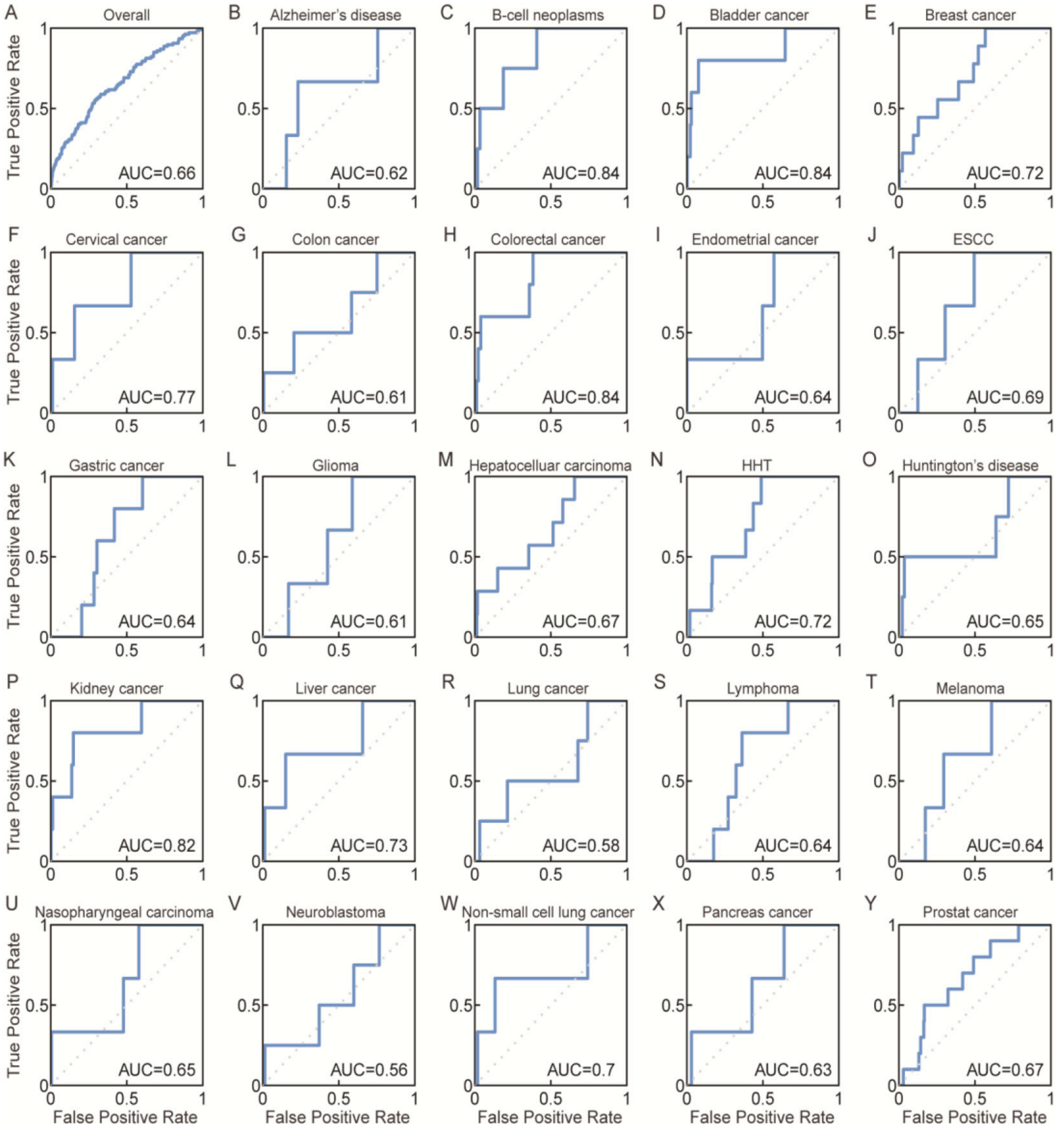

Supplementary Figure S7: ROC curves and AUC values for HyperTest method. (**A**) The overall ROC curve of HyperTest was 0.66. (**B**–**Y**) Case studies for 24 complex diseases in LOOCV analysis. HTT: Hereditary Haemorrhagic Telangiectasia.

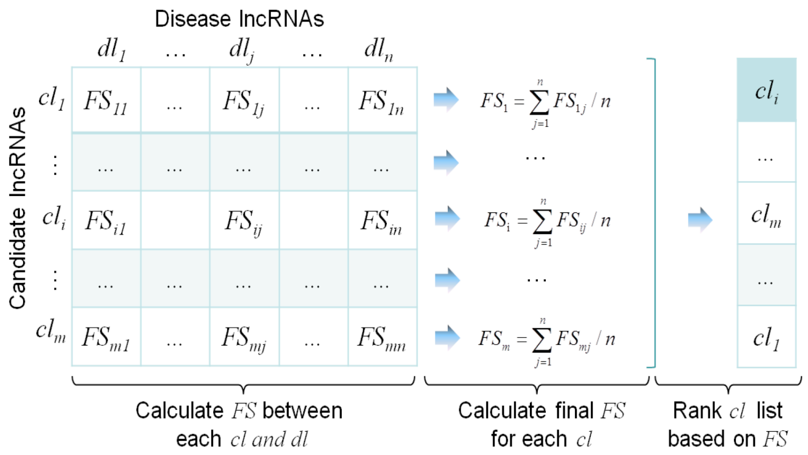

Supplementary Figure S8: Detailed information on methodology to calculate final FS score to prioritize candidate lncRNAs. For a given disease with n known disease lncRNAs($dl1$,... ,dlj,...,dln) and a set of m candidate lncRNAs ($cl1$,... ,cli,...,cln), the FS values between each cl and dl pair were calculated. For each of the candidate lncRNAs, n FS were generated and the average value of these n FS was calculated as final FS score. Further, the candidate lncRNAs were ranked based on the final FS. *cl*: candidate lncRNA, *dl*: disease lncRNA.

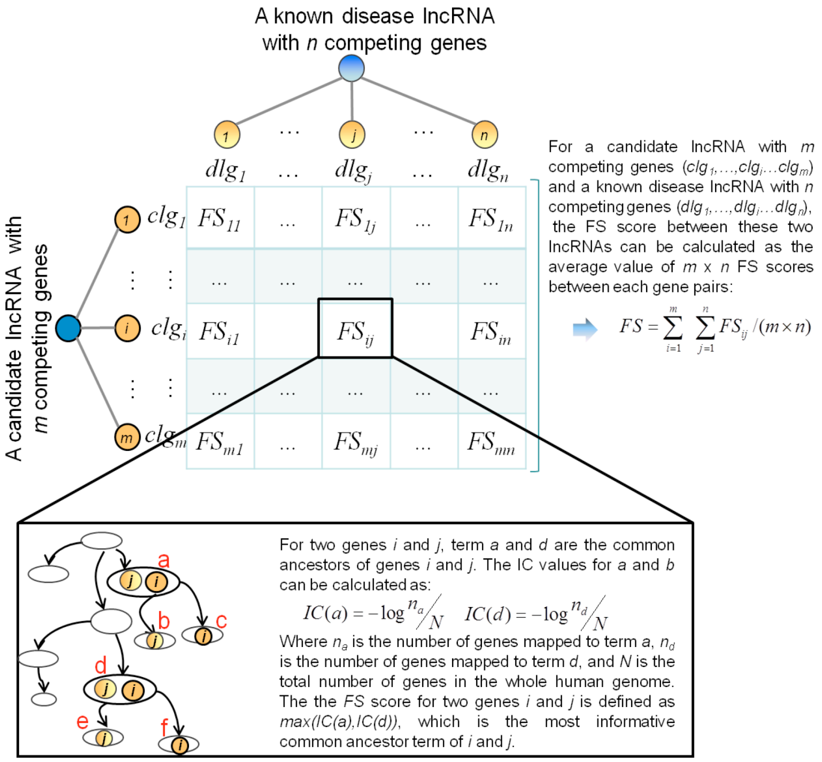

Supplementary Figure S9: Detailed information on the methodology used to decide the most informative common ancestor for two gene sets and how to calculate the FS between two lncRNAs based on GO context. *clg*: gene competed by a candidate lncRNA, *dlg*: gene competed by a disease lncRNA

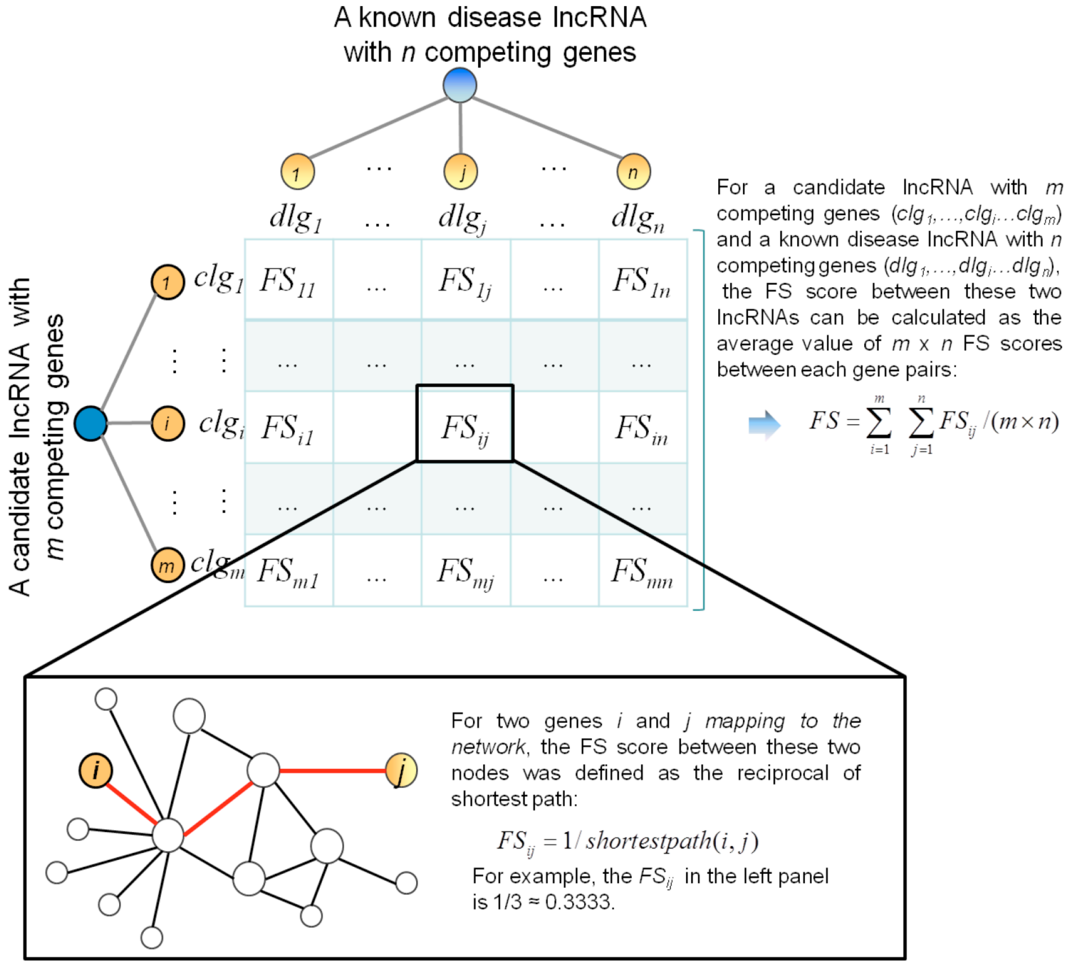

Supplementary Figure S10: Detailed information on methodology to calculate the final FS score for a candidate lncRNA based on the biological network context. For a candidate lncRNA, the FS score was calculated between the candidate and each known diseased lncRNA. *clg*: gene competed by a candidate lncRNA, *dlg*: gene competed by a disease lncRNA.

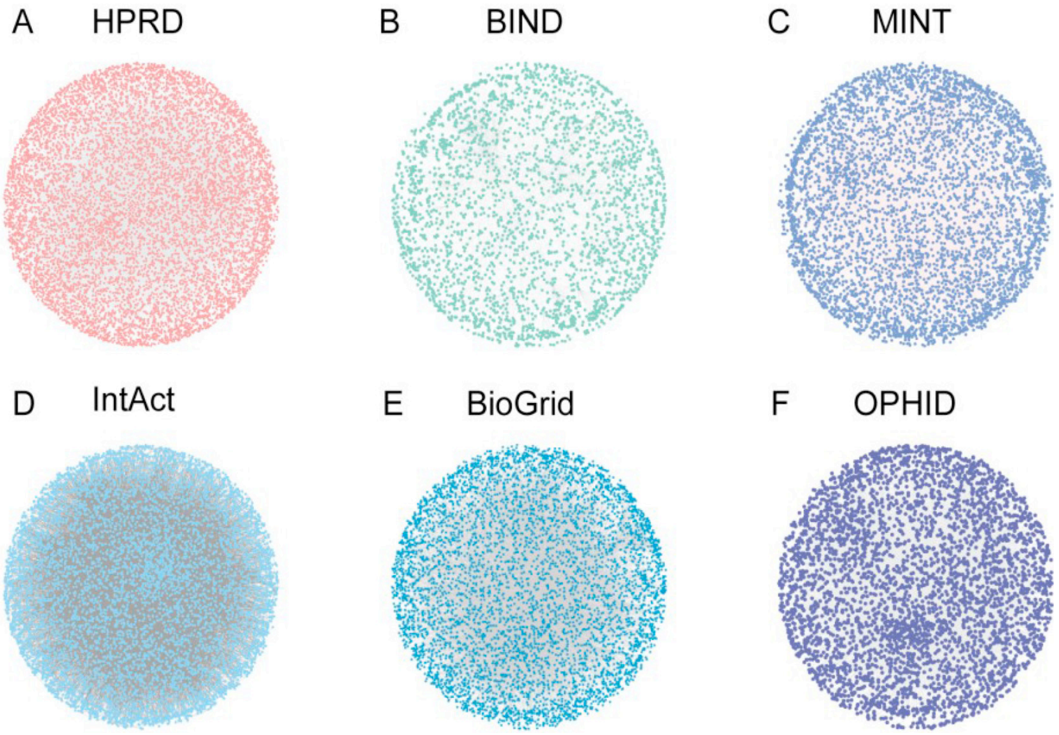

Supplementary Figure S11: A global view of the six biological networks based on Cytoscape software. In each network (**A**–**F**) genes were illustrated as nodes and interactions were illustrated as edges between different nodes.

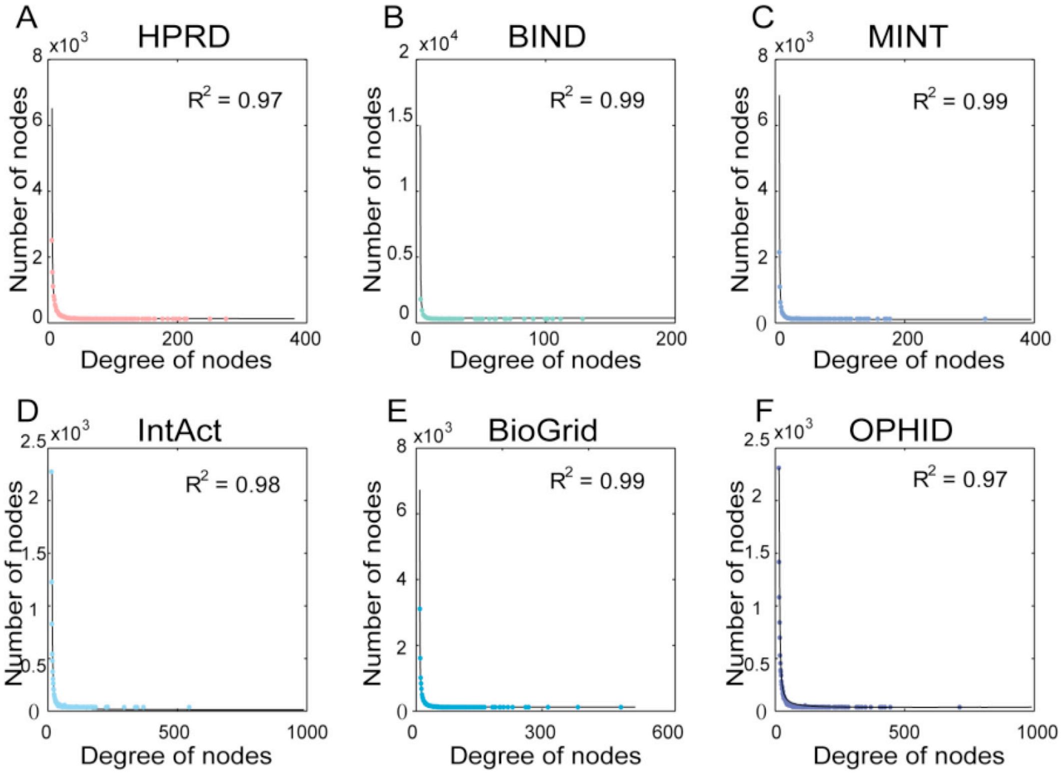

Supplementary Figure S12: An investigation of the nodes degree property revealing the power law distributions and the scale-free property of the biological networks (A–F).

Supplementary Table S1: Novel lncRNA-disease associations confirmed by literature survey in the ranked list from DisLncPri and other methods. see Supplementary_Table_S1

Supplementary Table S2: Nodes and interactions of six biological networks

	HPRD	BIND	MINT	IntAct	BigGrid	OPHID
Nodes	9048	3307	5028	5208	10030	11377
Interactions	36687	6076	13349	14342	39927	68989
