# Supplementary material for: Improved method for prioritization of disease associated lncRNAs based on ceRNA theory and functional genomics data
Source: Oncotarget. 2016 Dec 15;8(3):4642–55. doi: 10.18632/oncotarget.13964 (PMC5354861; doi:10.18632/oncotarget.13964)
Supplement: Supplementary file 2 [file oncotarget-08-4642-s002.docx]

**Table S1**. Novel lncRNA-disease associations confirmed by literature survey in the ranked list from DisLncPri and other methods. The total number of lncRNAs for each method are shown in the first line. The other numbers indicate the rank position and rank ratio of each lncRNA resulting from different methods. Top 10% ranked lncRNAs are listed.

| Diseases | LncRNAs | DisLncPri (1471) | | ExpSim (818) | | GaussSim (195) | | FunSim (1482) | | HyperTest (1481) | | Evidences (PubMed) |
| --- | --- | --- | --- | --- | --- | --- | --- | --- | --- | --- | --- | --- |
|  | MEG3 | 1 | (6.80E-04) | 2 | (2.44E-03) | 20 | (1.03E-01) | 52 | (3.51E-02) | 725 | (4.90E-01) | 22503900 |
| Alzheimer's disease | PVT1 | 6 | (4.08E-03) | 57 | (6.97E-02) | 10 | (5.13E-02) | 60 | (4.05E-02) | 594 | (4.01E-01) | 17503467,23990414 |
|  | LINC01616 | 13 | (8.84E-03) | 284 | (3.47E-01) |  |  | 95 | (6.41E-02) | 563 | (3.80E-01) | [26318290](http://www.ncbi.nlm.nih.gov/pubmed/26318290) ,21383047 |
|  | GAS5 | 1 | (6.80E-04) | 22 | (2.69E-02) | 22 | (1.13E-01) | 326 | (2.20E-01) | 96 | (6.48E-02) | 26503132 |
|  | MALAT1 | 4 | (2.72E-03) | 84 | (1.03E-01) | 20 | (1.03E-01) | 36 | (2.43E-02) | 60 | (4.05E-02) | 24379988 |
|  | MEG3 | 6 | (4.08E-03) | 7 | (8.56E-03) | 26 | (1.33E-01) | 250 | (1.69E-01) | 878 | (5.93E-01) | 24859196 |
| Ovarian cancer | HOTAIR | 9 | (6.12E-03) | 42 | (5.13E-02) | 12 | (6.15E-02) | 16 | (1.08E-02) | 580 | (3.92E-01) | 24662839 |
|  | ANRIL | 27 | (1.84E-02) | 105 | (1.28E-01) | 23 | (1.18E-01) | 176 | (1.19E-01) | 421 | (2.84E-01) | 25845387 |
|  | MNX1-AS1 | 53 | (3.60E-02) | 109 | (1.33E-01) | 16 | (8.21E-02) | 936 | (6.32E-01) | 21 | (1.42E-02) | 24379988 |
|  | OVAL | 71 | (4.83E-02) | 149 | (1.82E-01) | 21 | (1.08E-01) | 852 | (5.75E-01) | 16 | (1.08E-02) | 24265805 |
|  | GAS5 | 4 | (2.72E-03) | 6 | (7.33E-03) | 35 | (1.79E-01) | 36 | (2.43E-02) | 122 | (8.24E-02) | 24026436 |
|  | AP000221.1 | 11 | (7.48E-03) | 15 | (1.83E-02) | 32 | (1.64E-01) | 58 | (3.91E-02) | 4 | (2.70E-03) | 25755691 |
|  | CTC-338M12.5 | 17 | (1.16E-02) | 140 | (1.71E-01) | 4 | (2.05E-02) | 81 | (5.47E-02) | 496 | (3.35E-01) | 25755691 |
| Pancreatic cancer | HULC | 25 | (1.70E-02) | 256 | (3.13E-01) | 1 | (5.13E-03) | 92 | (6.21E-02) | 183 | (1.24E-01) | 20423907,25412939 |
|  | RP11-58D2.1 | 61 | (4.15E-02) | 71 | (8.68E-02) | 19 | (9.74E-02) | 50 | (3.37E-02) | 83 | (5.60E-02) | 25755691 |
|  | HOTTIP | 66 | (4.49E-02) | 417 | (5.10E-01) | 18 | (9.23E-02) | 191 | (1.29E-01) | 907 | (6.12E-01) | 25912306 |
|  | PVT1 | 92 | (6.25E-02) | 45 | (5.50E-02) | 5 | (2.56E-02) | 358 | (2.42E-01) | 636 | (4.29E-01) | 21316338,25668599 |
|  | FRGCA | 1 | (6.80E-04) | 180 | (2.20E-01) | 5 | (2.56E-02) | 34 | (2.29E-02) | 2 | (1.35E-03) | 26261500 |
|  | MALAT1 | 13 | (8.84E-03) | 197 | (2.41E-01) | 4 | (2.05E-02) | 44 | (2.97E-02) | 18 | (1.22E-02) | 24857172 |
|  | MEG3 | 20 | (1.36E-02) | 134 | (1.64E-01) | 76 | (3.90E-01) | 119 | (8.03E-02) | 452 | (3.05E-01) | 26253106 |
|  | GAS5 | 27 | (1.84E-02) | 284 | (3.47E-01) | 57 | (2.92E-01) | 109 | (7.35E-02) | 227 | (1.53E-01) | 24884417 |
|  | GS1-5L10.1 | 29 | (1.97E-02) | 52 | (6.36E-02) | 6 | (3.08E-02) | 172 | (1.16E-01) | 208 | (1.40E-01) | 26045391 |
|  | HIF1A-AS2 | 30 | (2.04E-02) | 700 | (8.56E-01) | 10 | (5.13E-02) | 88 | (5.94E-02) | 427 | (2.88E-01) | 25686741,9923855 |
|  | RP11-528G1.2 | 44 | (2.99E-02) | 594 | (7.26E-01) | 59 | (3.03E-01) | 72 | (4.86E-02) | 28 | (1.89E-02) | 26045391 |
| Gastric cancer | linc-ROR | 54 | (3.67E-02) | 111 | (1.36E-01) | 12 | (6.15E-02) | 69 | (4.66E-02) | 50 | (3.38E-02) | 26169368 |
|  | HNF1A-AS1 | 69 | (4.69E-02) | 674 | (8.24E-01) | 36 | (1.85E-01) | 156 | (1.05E-01) | 154 | (1.04E-01) | 26472090 |
|  | AC104699.1 | 76 | (5.17E-02) | 312 | (3.81E-01) | 45 | (2.31E-01) | 255 | (1.72E-01) | 437 | (2.95E-01) | 26045391 |
|  | RP11-789C1.1 | 86 | (5.85E-02) | 723 | (8.84E-01) | 56 | (2.87E-01) | 178 | (1.20E-01) | 374 | (2.53E-01) | 26045391 |
|  | nc886 | 113 | (7.68E-02) |  |  | 83 | (4.26E-01) | 157 | (1.06E-01) | 639 | (4.31E-01) | 25003254 |
|  | MYLK-AS1 | 119 | (8.09E-02) | 386 | (4.72E-01) | 67 | (3.44E-01) | 108 | (7.29E-02) | 186 | (1.26E-01) | 26045391 |
|  | LINC00982 | 121 | (8.23E-02) | 412 | (5.04E-01) | 114 | (5.85E-01) | 187 | (1.26E-01) | 57 | (3.85E-02) | 26334618 |
|  | RP11-643M14.1 | 127 | (8.63E-02) | 110 | (1.34E-01) | 42 | (2.15E-01) | 286 | (1.93E-01) | 63 | (4.25E-02) | 26045391 |
|  | BC040587 | 136 | (9.25E-02) | 105 | (1.28E-01) | 35 | (1.79E-01) | 400 | (2.70E-01) | 226 | (1.53E-01) | 25765901 |
